# Complete Genome Sequence of Arachnia rubra Strain DSM 100122^T^, a Cultured Member of the Human Oral Microbiome

**DOI:** 10.1128/MRA.00959-21

**Published:** 2021-12-02

**Authors:** Matthew Ouellette, Andrew A. Bejian, Tsute Chen, Dakota S. Jones, Christopher D. Johnston, Floyd E. Dewhirst

**Affiliations:** a The Forsyth Institute, Cambridge, Massachusetts, USA; b Department of Oral Medicine, Infection, and Immunity, Harvard School of Dental Medicine, Boston, Massachusetts, USA; c Vaccine and Infection Disease Division, Fred Hutchinson Cancer Research Center, Seattle, Washington, USA; University of Maryland School of Medicine

## Abstract

We report the complete genome of Arachnia rubra strain DSM 100122^T^. The genome is 3.32 Mb, with a GC content of 64.2%. The genome contains 3,005 predicted genes, including 2,923 predicted protein-coding genes.

## ANNOUNCEMENT

**FIG 1:**
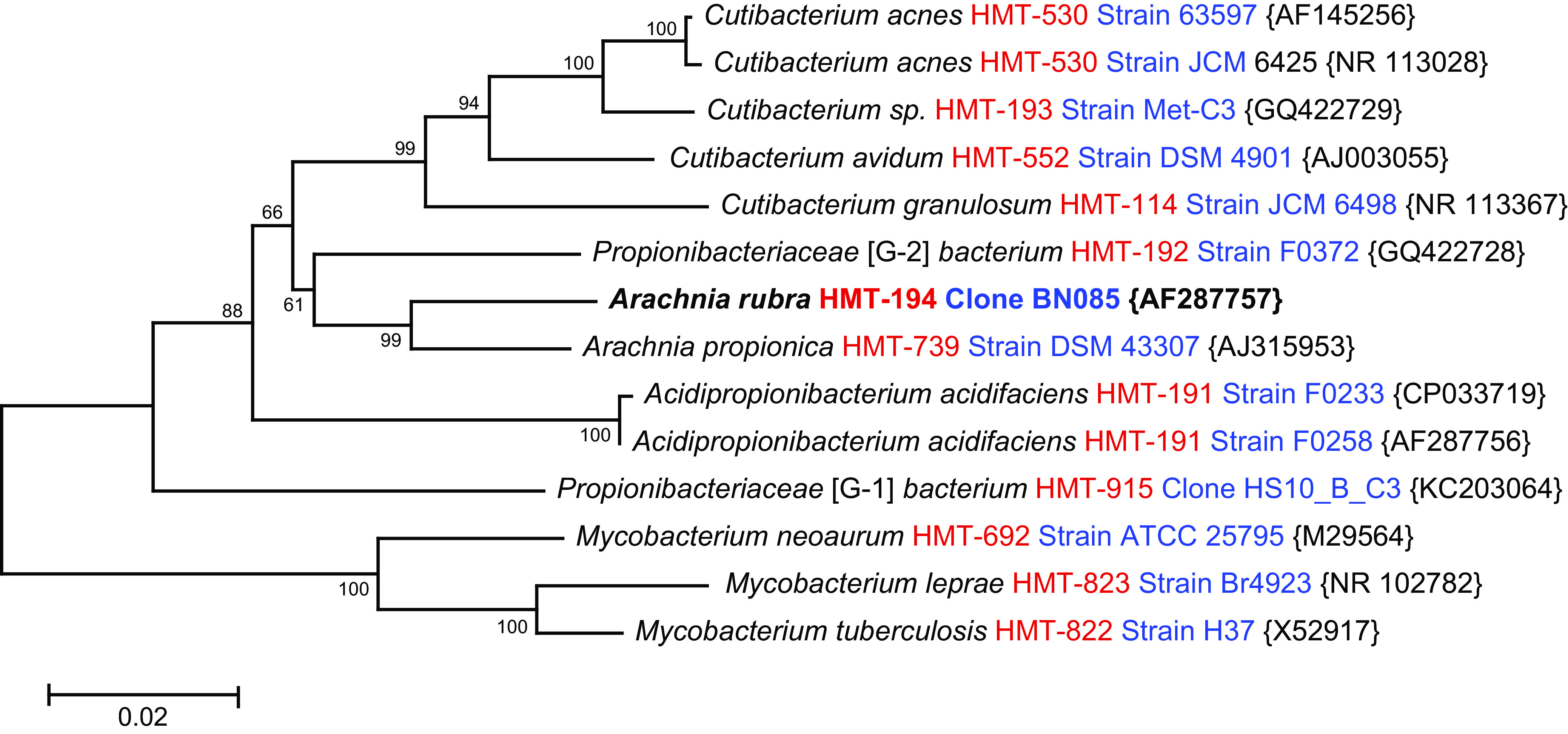
Neighbor-joining tree (13) for oral isolates of *Propionibacteriaceae*, with human oral Mycobacterium species included as an outgroup. Arachnia rubra strain DSM 100122^T^ is highlighted in bold. This tree was constructed in MEGA X (14) using aligned full-length 16S rRNA sequences (∼1,450 bp) downloaded from the Human Oral Microbiome Database (HOMD) (5, 15, 16). The evolutionary distances were computed using the Jukes-Cantor method (17) and are in the units of the number of base substitutions per site. The scale bar represents 0.02 base substitutions per site. Bootstrap support values for 1,000 replicates are indicated for each branch (18). GenBank accession numbers for 16S rRNA are provided in curly brackets.

Species within the genus *Propionibacterium* were recently divided into four genera, i.e., *Propionibacterium, Acidipropionibacterium*, *Cutibacterium*, and *Pseudopropionibacterium* (1). The name *Pseudopropionibacterium* was taxonomically corrected to *Arachnia* because it was a homotypic synonym (2). Arachnia propionica (3) and Arachnia rubra (4) are the only two recognized species in the genus *Arachnia*, and both are members of the human oral microbiome (5). A 16S rRNA neighbor-joining tree for oral species within the family *Propionibacteriaceae* with current taxonomy is shown in Fig. 1. Both *Arachnia* species are hosts for species of the phylum *Saccharibacteria* (TM7), ultrasmall parasitic epibionts (6–8). Several strains of *Saccharibacteria* species HMT-488 and HMT-955 have been grown in coculture with both A. propionica and A. rubra (8), and their genomes are listed under BioProject accession number 282954 (9, 10).

To fully examine the interactions of *Saccharibacteria* species with *Arachnia* hosts, it would be useful to have a genetically tractable strain of A. rubra and use it as a model host. Restriction modification (RM) systems are a major barrier to genetic transformation, and RM systems can be identified from the methylome obtained during single-molecule real-time (SMRT) genome sequencing (11). Based on the methylome data, plasmid vectors can be modified to eliminate RM incompatibilities with the target species, using techniques such as construction of syngenic DNA (12). The methylome reported here should facilitate efforts to make Arachnia rubra strain DSM 100122^T^ genetically tractable.

Strain DSM 100122^T^ was acquired from the German Collection of Microorganisms and Cell Cultures (DSM). For DNA isolation, the strain was grown in a 50:50 mixture of Trypticase soy broth and brain heart infusion broth with 1% yeast extract. Genomic DNA was extracted using a MasterPure DNA isolation kit (Lucigen) with a modified protocol that included bead beating for cell lysis. SMRT sequencing was carried out on a Sequel instrument (Pacific Biosciences, Menlo Park, CA, USA) with v3 chemistry, following standard SMRTbell template preparation protocols for base modification detection. Genomic DNA samples (5 to 10 μg) were sheared to an average size of 15 kbp via g-TUBE (Covaris, Woburn, MA, USA), end repaired, and ligated to hairpin-barcoded adapters prior to sequencing. Finally, prior to sequencing, the SMRTbell library was purified and size selected using AMPure PB beads to remove <3-kbp templates. Sequencing reads were processed using the Pacific Biosciences SMRT Link pipeline v8 (https://www.pacb.com/support/software-downloads) with Microbial Assembly under default parameters. A total of 145,877 subreads were obtained, covering 632,860,315 subread bases, with a mean read length of 4,329 bp and a read *N*_50_ value of 4,635 bp. The mean depth of coverage across the genome was 185×. A single circular contig of 3,316,958-bp length was assembled. The genomic GC content was 64.2%. The genome was annotated with the NCBI Prokaryotic Genome Annotation Pipeline (PGAP). A total of 3,005 genes were identified, including 2,923 predicted protein-coding genes, 56 predicted RNAs, and 26 predicted pseudogenes. Three motifs were identified as methylated throughout the genome, i.e., CTGCA^m6^G (2,690 modified motifs), ACGA^m6^BCT (2,130 modified motifs), and GAAA^m6^TG (712 modified motifs). REBASE analysis assigned the type II methyltransferase M.Aru100122I as being responsible for the CTGCA^m6^G motif modification, while the remaining modifications could not be assigned unambiguously to the remaining methyltransferase identified within the genome. Additionally, the genome harbors open reading frames (Aru100122McrBC) consistent with an active type IV restriction system, which should be taken into consideration during genetic engineering.

### Data availability.

The genome sequence was deposited in GenBank under the accession number CP072384 and SRA accession number SRR15979320. Base modification files were submitted with the GenBank submission, and the methylome analysis is available at REBASE with organism number 46978 for strain DSM 100122^T^. The BioProject accession number for this genome, as well as those of many other oral bacteria, is PRJNA282954.
